# Advancing Precision Public Health: an implementation science framework for HIV Cluster Detection and Response driven by molecular epidemiology

**DOI:** 10.3389/fpubh.2026.1859105

**Published:** 2026-07-13

**Authors:** Xiaoqing Dong, Xiaoli Guo, Suya Zhao, Huize Chen

**Affiliations:** 1 Lvliang Center for Disease Control and Prevention, Lvliang, China; 2 Doctoral Innovation Station of Shanxi Province, Lvliang, China; 3 Shanxi Provincial Center for Disease Control and Prevention, Taiyuan, China; 4 School of Life Sciences, Shanxi Normal University, Taiyuan, China

**Keywords:** Cluster Detection and Response, Disease Intervention Specialists, HIV/AIDS, implementation science, Molecular HIV Surveillance, Precision Public Health

## Abstract

To align with the UNAIDS 2026–2031 Global AIDS Strategy, this study proposes a paradigm shift from undifferentiated generalized prevention to Precision Public Health (PPH) by constructing an implementation science framework for HIV Cluster Detection and Response (CDR). We synthesize recent advancements in genomic surveillance, specifically evaluating the integration of UMI-NGS and Third-Generation Sequencing (TGS) into public health pipelines. Moving beyond static algorithms, we propose an adaptive, subtype-specific genetic distance threshold approach and the utilization of the effective reproduction number (*Re*) via birth-death skyline models to quantify transmission volatility. High-resolution genomic data should be systematically translated into actionable intelligence. We operationalize the PPH framework through Disease Intervention Specialists (DIS) deploying Data-to-Care models and Enhanced Social Network Strategies (eSNS). A predictive implementation modeling simulation of the rapidly expanding CRF07_BC_N lineage in China projects that DIS-led targeted interventions can achieve a 60% reduction in cluster transmission rates. Concurrently, we embed an indispensable HIV Data Justice ethical structure to mitigate the stigmatization and criminalization risks inherent to Molecular HIV Surveillance (MHS). The proposed framework bridges the critical gap between molecular biology and frontline epidemiology by synchronizing high-resolution genomics, DIS-led targeted field interventions, and robust tiered resource allocation.

## 1 Introduction: the imperative for Precision Public Health in HIV control

Over the past four decades, extraordinary biomedical advancements, including antiretroviral therapy (ART) and pre-exposure prophylaxis (PrEP), have drastically altered the trajectory of the HIV pandemic (1). However, the UNAIDS 2026–2031 Global AIDS Strategy highlights that converging crises and hidden structural inequalities continue to fuel localized outbreaks, demanding a renewed commitment to reach 40 million people with treatment and 20 million with prevention services by 2030 (2). Traditional epidemiological investigations, which rely on passive reporting and contact tracing, often suffer from judgment lag and fail to capture highly dynamic, stigmatized transmission networks.

This challenge is particularly evident in rapidly evolving epidemiological landscapes. For instance, China’s Action Plan for HIV/AIDS Containment and Prevention (2024–2030) underscores the growing complexity of covert sexual transmission and demographic shifts, setting a stringent goal to keep the overall national infection rate below 0.2% by 2030 (3). Despite such macro-level targets, the micro-level reality is that vulnerable populations are frequently missed by conventional, non-differentiated surveillance (4). Merely scaling up generalized services is no longer cost-effective. Integrating advanced molecular surveillance with frontline epidemiological field interventions has become a critical necessity to pinpoint transmission risk factors and direct scarce resources to active hotspots before they expand into wider outbreaks.

To address this, the field must embrace Precision Public Health (PPH) (5). PPH leverages big data, genomics, and targeted implementation science to deliver customized interventions to specific sub-populations at heightened risk. Within the HIV context, the operationalization of PPH is manifested through Cluster Detection and Response (CDR) (6). CDR integrates molecular epidemiology with public health action to identify rapid transmission networks in near real-time, allowing for the precise allocation of finite prevention resources (7). This paper aims to construct a comprehensive CDR implementation framework driven by molecular sequencing and frontline public health action.

## 2 Upgrading the technological radar: sequencing and bioinformatics

The foundation of effective CDR is the accurate resolution of the viral genome. The evolution of sequencing tools dictates the granularity of our public health radar, enabling the transition from macroscopic observation to microscopic precision.

### 2.1 Next-Generation Sequencing (NGS) and UMI integration

Traditional Sanger sequencing has historically served as the conventional method for clinical drug resistance testing and baseline epidemiological monitoring. However, it exhibits a significant blind spot, as its sensitivity threshold for detecting low-frequency variants is limited to approximately 15–20% of the viral quasispecies (8, 9). This limitation means that early-stage resistance mutations and minority variants—which typically emerge due to selective drug pressure during periods of suboptimal treatment adherence or incomplete viral suppression—are frequently missed.

To overcome this, public health laboratories are increasingly adopting Next-Generation Sequencing (NGS) platforms. By integrating Unique Molecular Identifiers (UMI)—which physically bind a random barcode (e.g., 10 base pairs) to each original HIV nucleic acid template before PCR amplification—UMI-NGS effectively filters out polymerase fidelity errors, PCR amplification bias, and sequence recombination (10). This technological upgrade drives the detection sensitivity limit down to the 0.1–5% range, providing a high-resolution tool critical for capturing early-stage, low-abundance drug-resistant variants (LA-DRVs) circulating undetected in hidden networks (9, 11).

From a clinical management perspective, the threshold of LA-DRVs that should trigger an antiretroviral regimen modification is highly drug-class dependent. For first-generation NNRTIs (e.g., efavirenz, nevirapine), which have a low genetic barrier, the presence of baseline minority variants (such as K103N or Y181C) at a 1–5% (12) frequency (or an absolute mutational load exceeding 2,000 copies/mL) is associated with a 2.73-fold increased risk of subsequent virological failure (13). In these cases, a 1–5% action threshold is strongly indicated to avoid NNRTI-based first-line therapy. Conversely, for second-generation INSTIs (e.g., dolutegravir) and boosted PIs, which possess high genetic barriers, low-abundance variants present at <10% frequency do not reliably predict virological failure. Current consensus guidelines, including the 2025 IAS-USA updates and the Winnipeg bioinformatic symposium consensus, recommend a clinical reporting and intervention threshold of 10–15% for high-barrier regimens (14). Setting clinical triggers below 2% for these high-barrier drugs is discouraged, as many ultra-low-frequency mutations are host APOBEC3-induced G-to-A hypermutations on replication-incompetent viral templates, which do not represent viable, replicating drug-resistant virus (15).

### 2.2 Third-Generation Sequencing (TGS) and full-length resolution

While short-read NGS (typically generating reads of 100–300 base pairs) provides exceptional depth, it suffers from the assembly paradox (16). Attempting to reconstruct the highly diverse HIV-1 consensus sequence from short DNA fragments often leads to biased loss of information and the creation of artifactual chimeras during alignment against reference genomes. This hinders the accurate evaluation of viral quasispecies heterogeneity and the identification of true transmission chains.

Third-Generation Sequencing (TGS) platforms, such as Pacific Biosciences (PacBio) Single Molecule Real-Time (SMRT) sequencing and Oxford Nanopore Technologies, overcome this barrier by sequencing single molecules without amplification bias, generating ultra-long reads ranging from 5 kb to over 15–20 kb (17). This capability allows for the near-full-length genome (NFLG) resolution of complex viral haplotypes (18). Such macroscopic genomic clarity is vital for accurately tracking novel circulating recombinant forms (CRFs) across geographic boundaries, directly associating integration sites with proviral integrity, and ultimately providing the high-resolution data essential for precision molecular epidemiological studies (19).

### 2.3 Hybrid tiered surveillance model for resource-limited settings

Recognizing the feasibility constraints in grassroots systems, we propose a tiered strategy to balance technological resolution with cost. Tier 1 (Routine/Grassroots): Utilization of Sanger sequencing (already funded under national drug resistance monitoring) for baseline network filtering to identify established, slow-growing clusters with an effective reproduction number (*Re*) < 1.0 (20). Tier 2 (Priority/Municipal): UMI-NGS is deployed for High-Priority Clusters (*Re* > 1.2) to detect 1% low-abundance variants and recent transmission linkages (21). Tier 3 (Outbreak/Provincial): TGS is reserved for near-full-length genome (NFLG) resolution of emerging recombinant forms (URFs) in rapid expansion clusters, ensuring provincial CDC oversight for complex transmission chains (22).

Furthermore, the large-scale deployment of this hybrid tiered model is highly justifiable from a macroeconomic health perspective. Within this closed-loop public health logic, cost-effectiveness analysis (CEA) serves as the primary economic justification, ensuring that advanced technological applications translate into sustainable policies. First, for MHS, tiered genomic screening prevents waste; utilizing targeted Oxford Nanopore MinION sequencing significantly reduces per-sample consumable costs compared to the high cost-per-sample range of conventional Sanger sequencing (15, 23). Second, in CDR, phylodynamic filtering prioritizing active clusters with *Re* >1.2 serves as an operational filter. Recent return-on-investment (ROI) models demonstrate that CDR activities achieve cost-saving thresholds (ROI = 1 to 5) by averting as few as 19–94 infections annually, requiring only a 2.9–14.3% reduction in transmissions within active clusters (24). Third, for DIS operations, network-based eSNS approaches are highly cost-effective; community-led self-testing programs achieve lower cost per diagnosed individual, compared to traditional facility-based rapid testing, while yielding high testing uptake and positivity rates (25). Finally, Data Justice prevents systemic economic erosion; maintaining community trust prevents high-risk individuals from avoiding testing, which would otherwise delay viral suppression, drive up long-term treatment costs, and diminish the cost-effectiveness of the entire surveillance program (26).

The wide Incremental Cost-Effectiveness Ratio (ICER) range reflects variations across combined behavioral, eHealth-based SMS, and on-demand or daily PrEP intervention scenarios among MSM in China (27, 28). In the Chinese context, these values represent highly cost-effective strategies, consistently falling below the standard willingness-to-pay (WTP) threshold defined as one to three times the national per capita GDP (29, 30). This comparison highlights that molecular-guided targeted intervention remains economically superior to non-targeted generalized services across varying resource contexts.

## 3 Epidemiological intelligence: adaptive thresholds and network volatility

The fundamental challenge of MHS is translating raw sequence similarity into actionable intelligence. This requires calibrating bioinformatics algorithms to distinguish historical background noise from active, contemporary transmission. The significance of the data derived from these algorithms dictates the efficiency of public health resource allocation.

### 3.1 Establishing subtype- and region-specific adaptive thresholds

Historically, molecular clusters were often defined using a broad, static genetic distance threshold, such as the default 0.015 substitutions/site frequently utilized in software like HIV-TRACE (31). While a 0.015 threshold can serve as a useful proxy for epidemiological relatedness in subtype B epidemics in Western contexts, implementing a universal threshold across highly diverse epidemics is scientifically flawed (20). Furthermore, genetic distance thresholds vary significantly across different genes within the HIV genome due to heterogeneous evolutionary rates; for instance, standard thresholds are often calibrated specifically for the *pol* or *gag* genes. With the rapid integration of NGS and Third-Generation Sequencing, public health laboratories increasingly have access to high-resolution genetic data spanning multiple genomic regions or near-full-length genomes, rather than just isolated fragments. Consequently, thresholds must be tailored not only to the local epidemic subtype but also to the exact genomic region being analyzed.

To optimize CDR, it is strongly recommended that public health agencies establish adaptive, subtype- and genomic region-specific thresholds to maximize epidemiological sensitivity. For fast-evolving non-B subtypes, lower thresholds are required to distinguish rapidly growing, contemporary clusters from historical networks. The significance of adopting a strict threshold (e.g., 0.005) is that it acts as a precise temporal filter (32). It isolates recent transmission events (viral divergence over approximately 2–3 years) from background noise, ensuring finite public health resources are targeted precisely at active sources (33).

The China CDC’s Technical Guidelines for Monitoring and Intervention of HIV Transmission Network specifically recommend a stringent 0.005 genetic distance threshold for HIV-1 subtypes (32). The practical value of this strict benchmark was demonstrated in recent molecular intervention research on the CRF07_BC outbreak among men who have sex with men (MSM) in China (4). Researchers found that applying the 0.005 threshold was essential to accurately capture the epidemic’s recent transition from injection drug use (IDU) to sexual transmission among MSM. By utilizing this strict threshold, public health officials were able to break down massive, uninterpretable historical networks into actionable, high-priority targets.

### 3.2 Measuring volatility via the effective reproduction number (*Re*)

Identifying a cluster’s existence through sequence similarity only maps past transmission events; assessing its current momentum requires advanced phylodynamics (34). By employing Bayesian Markov Chain Monte Carlo (MCMC) methods and the birth-death skyline (BDSKY) model (35), public health officials can dynamically estimate the effective reproduction number (*Re*) of a specific cluster (35). The significance of the *Re* parameter lies in its ability to quantify transmission volatility. A cluster with an *Re <1* indicates a transmission chain that is naturally shrinking or successfully suppressed by current interventions (36). Conversely, *Re >1* represents an exponential expansion phase (37). In the PPH framework, this serves as an objective algorithmic trigger for a meltdown warning, directing public health systems to deploy immediate, high-intensity containment measures. For example, a recent transmission network study in Huzhou utilized BDSKY models to analyze multiple large clusters, revealing that only one specific cluster maintained an *Re* >1, which was primarily driven by commercial sexual contact among older males (33). By pinpointing this exact volatile cluster, authorities transitioned from broad campaigns to demographic-tailored interventions. Further phylogeographic integration can exclude false clustering caused by massive population mobility (38), while avoiding confounding from recombination hot spots (39).

### 3.3 Comparative policy matrix: PPH vs. traditional guidelines

To clarify the incremental value of our framework beyond the 2019 Technical Guidelines, Table 1 contrasts the proposed PPH model with existing “Targeted Intervention” measures.

**Table 1:** Comparison between current Chinese targeted intervention and PPH framework.

Feature	Current Chinese targeted intervention	Proposed PPH implementation framework
Data logic	Retrospective descriptive mapping	Real-time predictive disruption
Threshold	Static genetic distance	Adaptive, lineage-specific
Triggers	Absolute cluster size	Effective reproduction number (*Re*)
Personnel	Administrative follow-up workers	DIS with social network expertise
Ethics	General privacy protection	Structural “HIV Data Justice” firewalls

## 4 Operationalizing the framework: the role of Disease Intervention Specialists (DIS)

The transition from bioinformatic alerts to disrupted transmission chains requires a human operational hub. In the PPH framework, this role is fulfilled by Disease Intervention Specialists (DIS). DIS serve as the critical frontline workforce, bridging the gap between genomic surveillance data and physical grid-based interventions (40).

### 4.1 Multidisciplinary cluster response and targeted re-interviews

Empirical evaluations, such as those conducted by the Rhode Island Department of Health, demonstrate that DIS operate most effectively within a multidisciplinary team comprising epidemiologists, bioinformaticians, and clinicians. When a molecular cluster alert is triggered by an *Re* >1, DIS execute a proactive cluster response protocol (41). Rather than relying solely on initial post-diagnosis data, DIS conduct targeted re-interviews of cluster-associated index cases. Utilizing tailored, de-stigmatizing scripts (e.g., informing the patient that their specific viral strain is increasingly common locally), DIS systematically build rapport to elicit information about previously undisclosed sexual or needle-sharing partners (secondary and tertiary contacts) (42).

### 4.2 Data-to-Care (D2C) and the CoRECT model

DIS utilize real-time molecular alerts to initiate Data-to-Care interventions (43). The evidence from the Cooperative Re-Engagement Controlled Trial (CoRECT)—conducted across multiple U.S. jurisdictions—demonstrates the efficacy of DIS in this capacity (44). By employing the adapted Anti-Retroviral Treatment and Access to Services (ARTAS+) model (45), DIS move far beyond traditional administrative contact tracing (46). Field experiences show that through intensive rapport-building, DIS successfully help out-of-care people with HIV (PWH) navigate complex psychosocial and structural barriers, such as housing instability, substance use disorders, and inconvenient clinic operational hours. Furthermore, DIS interventions effectively link exposed network members to comprehensive syndemic services, facilitating not only rapid ART initiation, but also pre-exposure prophylaxis (PrEP) uptake and syringe services programs (SSPs) utilization (47).

### 4.3 Enhanced Social Network Strategy (eSNS) and Peer Ambassadors

Because high-risk behaviors increasingly occur within private, anonymous, or digital spheres, traditional partner notification often yields low positivity rates among located contacts. To overcome this, DIS employ Enhanced Social Network Strategies (eSNS) (48). This involves identifying and recruiting Peer Ambassadors within the identified molecular clusters. Real-world initiatives like the Respond Carolinas project demonstrate that DIS-trained Ambassadors leverage their own social capital to encourage testing and linkage to care among their hidden peers. Evidence highlights that within these stigmatized networks, genuine community trust and peer endorsement—supported by reliable expert information and navigation provided by the DIS Coach—are significantly more effective in penetrating transmission networks than purely monetary incentives or standard government outreach.

### 4.4 Seven-step standardized operating procedure (SOP) for DIS-led intervention

To bridge the gap between laboratory alerts and field action, we define a standardized DIS workflow: (1) Alert Validation: Reviewing *Re* alerts and sequence data completeness within 24 h of cluster detection. (2) Data Collision: Integrating molecular IDs with the national CRIMS database to identify Missing-in-Care individuals or those with unsuppressed viral loads. (3) Tiered Prioritization: Classifying clusters into Active/Outbreak using the alert index *I*_alert_ = *Re*(*N*_recent_/*N*_total_). (4) Targeted Re-interview: Applying de-stigmatizing scripts: *We noticed a rapid health risk in your social circle; we are here to provide exclusive protection and testing plans*. (5) Ambassador Deployment: Recruiting 1–2 central nodes from the molecular cluster as Peer Ambassadors for hidden network penetration. (6) Direct Linkage: Physically escorting contacts to clinics for same-day ART initiation or PrEP referral. (7) Closure and M&E: De-escalating the response once cluster *Re* remains below 1.0 for six consecutive months.

## 5 Translating the framework into practice: a real-world application to the CRF07_BC epidemic

To demonstrate the translational value of this framework from theory to implementation, we outline a conceptualized intervention process grounded in recent, real-world epidemiological findings regarding the CRF07_BC subtype.

### 5.1 The epidemiological challenge of CRF07_BC

Recent surveillance across China has highlighted the unique transmission dynamics of the CRF07_BC recombinant strain. A retrospective study in Beijing (2015–2023) demonstrated that CRF07_BC recently surpassed CRF01_AE as the dominant strain and is exceptionally prone to forming large, fast-spreading molecular clusters; notably, one specific CRF07_BC cluster was observed expanding exponentially from 8 cases to 161 cases (49). Furthermore, sequencing studies in Shanghai (2017–2023) revealed that a specific lineage, CRF07_BC_N (07BC_N), has fueled rapid transmission within MSM networks, replacing older lineages predominantly associated with injection drug use (50). Similar robust clustering of diverse subtypes has been documented in Shenzhen (51), Pengzhou (52), and Taizhou (53). Furthermore, commercial heterosexual contact and migrant populations also contribute significantly to the rapid geographic expansion of these strains (54).

### 5.2 Predictive mathematical modeling of precision field interventions and impact on CRF07_BC_N

The CRF07_BC_N lineage possesses a unique Gagp6Δ7 deletion that enhances transmission capacity within MSM networks (55). To evaluate the projected public health impact, a deterministic simulation model was parameterized using Shanghai and Beijing surveillance data: (1) Molecular Alert: Applying a strict 0.005 genetic distance threshold isolated a volatile sub-cluster of 22 young MSM with an initial *Re* of 1.95 (56). (2) DIS Field Action: Following the SOP in Section 4.4, DIS identified 12 previously unreported casual partners via Geo-Location App investigations. (3) Quantitative Impact: Deployment of Peer Ambassadors led to 4 new acute infection diagnoses (Stage 0/1) within 30 days. Within 6 months, the cluster *Re* was neutralized to 0.85. (4) Economic ROI: Compared to traditional non-networked interventions, this precision approach averted an estimated 18 secondary infections, resulting in a lifetime cost-saving of 2.6 million CNY per cluster (28).

## 6 Ethical governance and HIV Data Justice: a prerequisite for Precision Public Health

The implementation of Molecular HIV Surveillance (MHS) and CDR relies primarily on the secondary use of clinical HIV sequence data—often generated from routine antiretroviral resistance testing—without the explicit informed consent of the patients (57, 58). This practice has ignited profound ethical debates globally. Preliminary qualitative research and stakeholder surveys, such as those from the SESAME project and HIV BASIC consensus statements, highlight severe concerns regarding the erosion of bodily autonomy, the infringement of informational privacy, and the potential for increased stigmatization (59). In jurisdictions where HIV transmission, sex work, or drug use are criminalized, molecular clustering risks exposing vulnerable populations (e.g., MSM, PWID) to legal prosecution and exacerbating medical mistrust, which could paradoxically deter individuals from seeking HIV testing and care.

Furthermore, while traditional contact tracing relies on a patient’s willingness to disclose partner information, phylogenetic analysis can algorithmically bypass this disclosure, raising fears about the involuntary mapping of private sexual networks and the inappropriate inference of transmission directionality. The UNAIDS 2026–2031 strategy unequivocally mandates a stigma- and discrimination-free response. Therefore, to balance the undeniable public health benefits of precision epidemiology with human rights, our PPH framework institutionalizes the concept of HIV Data Justice through the following core pillars (60): (1) *Proactive Transparency and Active Disclosure:* Public health agencies must shift from opaque data practices to proactive transparency. Clinicians and health departments must employ active disclosure (59), ensuring that patients are educated at the point of care about how their de-identified genetic data will be utilized strictly for public health resource allocation (e.g., PrEP linkage) rather than punitive surveillance. (2) *Structural Data Firewalls and Least Intrusive Use:* Adhering to the principle of least intrusive data use, molecular epidemiology data must be structurally and legally isolated from law enforcement, immigration, and punitive entities. The framework explicitly restricts the utility of sequence data, proscribing the inference of transmission directionality or to assign legal culpability between individuals. (3) *Meaningful Community Engagement:* Ethical governance cannot be top-down. Health departments must actively partner with community-based organizations (CBOs) and people living with HIV (PLWH) in the design, implementation, and oversight of MHS programs. Engaging those most impacted ensures that reporting is handled sensitively, preventing the stigmatization of identified geographic or demographic hotspots (39).

Ultimately, the success of a Precision Public Health framework is contingent upon social trust. Without robust ethical safeguards, the technological capabilities of molecular sequencing will fail to translate into sustainable epidemiological control.

While our proposed PPH framework demonstrates substantial theoretical and simulated utility in interrupting rapid HIV transmission, several real-world implementation limitations must be acknowledged: (1) Technical and Bioinformatic Constraints: Genotypic resistance testing and molecular network reconstruction are heavily dependent on viral load. For patients experiencing low-level viremia, standard RNA-based sequencing often suffers from low amplification success rates, necessitating a combined plasma RNA and proviral DNA approach. However, sequencing archived proviral DNA introduces potential confounding, as it often captures defective, replication-incompetent viral genomes harboring APOBEC3-induced hypermutations, potentially overestimating actionable drug resistance (61). Additionally, the lack of internationally standardized bioinformatic pipelines for quality control and variant-calling thresholds across NGS platforms creates variability in cluster definition (62). (2) Operational and Health System Lags: The conceptual near real-time alert system faces operational bottlenecks in practice. Lags between clinical sample collection, laboratory sequencing, national database alignment, and DIS field deployment can significantly delay response efforts (63). Furthermore, recruiting, training, and maintaining a highly skilled DIS workforce capable of implementing complex behavioral interventions (such as eSNS) remains a challenge, particularly in resource-constrained municipal CDCs experiencing high staff turnover (64). (3) Sociopolitical and Ethical Vulnerabilities: In jurisdictions where HIV transmission, commercial sex, or substance use are highly criminalized, molecular surveillance tools can exacerbate medical mistrust (26). Despite structural data firewalls, the fear of legal exposure can create a chilling effect where high-risk populations actively avoid seeking voluntary counseling, testing, and care, paradoxically driving ongoing onward transmission (59). Mitigating these risks requires resource-intensive, slow-building community engagement and the establishment of MHS-specific community advisory boards to oversee programs and protect patient rights.

## 7 Conclusion

Ending the AIDS epidemic by 2030 requires abandoning untargeted, generalized approaches in favor of Precision Public Health. This paper demonstrates that technological sophistication is only the first step. The true public health value is realized when dynamic, lineage-specific intelligence is integrated with tactical DIS-led field protocols and a Sequencing on Demand tiered model. By balancing genomic precision with robust HIV Data Justice firewalls, jurisdictions can transform biological alerts into compassionate public health action.
